# Drug addiction stigma in relation to methadone maintenance treatment by different service delivery models in Vietnam

**DOI:** 10.1186/s12889-016-2897-0

**Published:** 2016-03-08

**Authors:** Bach Xuan Tran, Phuong Bich Vu, Long Hoang Nguyen, Sophia Knowlton Latkin, Cuong Tat Nguyen, Huong Thu Thi Phan, Carl A. Latkin

**Affiliations:** Institute for Preventive Medicine and Public Health, Hanoi Medical University, Hanoi, vietnam; Department of Health, Behavior and Society, Johns Hopkins Bloomberg School of Public Health, Baltimore, MD USA; School of Medicine and Pharmacy, Vietnam National University, Hanoi, Vietnam; River Hill, Maryland, USA; Institute for Global Health Innovations, Duy Tan University, Da Nang, Vietnam; Authority of HIV/AIDS Control, Ministry of Health, Hanoi, Vietnam

**Keywords:** Stigma, Drug addiction, Methadone maintenance treatment

## Abstract

**Background:**

The rapid expansion of methadone maintenance treatment (MMT) services has significantly improved health status and quality of life of patients. However, little is known about its impacts on addiction-related stigma and associated factors.

**Methods:**

A cross-sectional survey was conducted in 2013 in Vietnam’s capital, Hanoi, and Nam Dinh province among 1016 methadone maintenance patients; 26.6 % at provincial AIDS centers (PAC) and 73.4 % at district health centers (DHC), respectively. Drug addiction history and related stigma, health status, MMT-related covariates, and sociodemographic characteristics were interviewed.

**Results:**

More than one-sixth of the sample reported experiencing felt or enacted stigma, including Blame or Judgement (17.2 %), Shame (19.9 %), or Others’ fear of HIV transmission (17.1 %). These proportions were higher in PACs than in DHCs, which are integrated with other HIV or general health care services. Very few patients reported being discriminated at the workplace (2.5 %) or at health care services (1.7 %); however, 15.6 % of patients at PACs and 10.6 % of patients at DHCs reported discrimination in their communities. Drug users taking MMT for longer periods were less likely to report felt stigma. Other factors associated with stigma against MMT patients included the lack of comprehensive services, higher education, presence of pain/discomfort, and anxiety/depression, self-reported HIV positive, and number of previous drug rehabilitation episodes.

**Conclusion:**

The study shows a high level of stigma against MMT patients and emphasizes the necessity to integrate MMT with comprehensive health and support services. Mass communication campaigns to reduce stigma against people with drug addiction and HIV/AIDS, as well as vocational trainings and jobs referrals for MMT patients, are needed to maximize the benefits of MMT programs in Vietnam.

## Background

Illicit drug use has been recognized as a major global public health issue and continues to drive HIV epidemics in various low and middle-income countries. In 2013, an estimation of 213 million people still used illicit drugs, with 27 million having health problems and approximately 1.65 million living with HIV making it one of the leading attributable factors to the global burden of disease [1]. In Vietnam, along with sex workers, people who inject drugs (PWID) have been labelled “social evils” due to their high prevalence of perceived immoral behaviors such as criminal activities or deteriorating health, which could threaten the safety of the population [2, 3].

Methadone is a highly effective medication for opioid dependence [4] and methadone maintenance treatment (MMT) has been found to improve health status and promote access to health care among drug users [5–7]. Moreover, MMT helps to reduce the frequency of illicit drug use [7–9], HIV-related risk behaviors [10, 11] and illegal activities [12, 13]. Expanding the coverage of MMT has a major role as a cost-effective strategy in planning HIV/AIDS prevention and control programs in both lower and upper-income countries [7, 14, 15].

However, drug users might confront stigmatization even when they enroll in MMT programs [14]. Those infected with HIV/AIDS may suffer from drug use and HIV stigma. Discrimination may occur at multiple locations, such as health care facilities and family, community, or work places. For example, a study of Ahern et al. showed that 75.2 % of drug users experienced discrimination in their family [16]. Stigma attached to drug use has been found to have negative effects on the health status of drug users and to hinder treatment adherence and health improvement [14, 17, 18]. Therefore, understanding influential factors and identifying strategies to reduce drug addiction-related stigma are essential for maximizing the effectiveness of MMT programs.

In Vietnam, PWID are one of the most-at-risk populations and account for about a half of the total number of people living with HIV/AIDS [6]. To respond, the government of Vietnam has developed comprehensive HIV/AIDS policies and programs, including a plan for expanding MMT programs to 80,000 drug users [19]. In 2015, there were only 170 MMT clinics nationwide with 31,200 patients [20, 21]. MMT service has been delivered in stand-alone or integrative models, which are co-located or managed with other HIV-related or general health care services. The MMT services are organized with trained specialists and standardized facilities following national guidelines established by the Vietnam Ministry of Health. There have been studies that examined the experiences of MMT patients and sources of stigma [14, 22–24] as well as the role of services providers. Very few studies, however, have focused on different service delivery models or have been conducted in the context of a large drug injection-related HIV epidemic. This study examines the differences in levels of felt and enacted stigma that MMT patients may experience across different service delivery models and levels of health administration.

## Methods

### Survey design and sampling

A cross-sectional survey was performed from June to August 2013 in Hanoi and Nam Dinh provinces. There were five MMT clinics involved, four of which were located at district health centres (DHC), namely Tu Liem, Ha Dong, Long Bien, and Xuan Truong, and one clinic was at Nam Dinh Provincial AIDS Center (PAC). The characteristics of study sites are listed in Table 1. We selected the two provinces in consultation with program managers at the Vietnam Authority of HIV/AIDS for a purposive comparison of an experienced setting, Hanoi, and a new setting, Nam Dinh Province. The MMT sites were primarily selected for the comparison of various service delivery models in different level of health administration. In general, drug users register at the nearest MMT clinics and these clinics provide treatment regardless of patients’ HIV status. In the organization of Vietnam’s health services delivery system, the regional polyclinic is providing primary health care for an area that combines several communes and is linked to commune health stations [25, 26]. Therefore, the criteria for enrolling drug users in selected MMT were indifferent.

**Table 1:** Study settings and sample size

Level	Settings	Site Name	Type of services	Sample size
Province	Nam Dinh City	Provincial AIDS Center	MMT+ VCT	270
District (rural)	Xuan Truong District	District Health Center	MMT+ VCT + ART + GH	151
District (urban)	Tu Liem District	District Health Center	MMT+ VCT + ART + GH	201
District (urban)	Long Bien District	District Health Center	MMT+ VCT + ART + GH	184
District (urban)	Ha Dong District	Regional Polyclinic	MMT+ GH	210

Eligibility criteria for recruiting participants included: 1) taking or initiating MMT in selected sites; 2) presenting at clinics during study periods; 3) being 18 years old or above; 4) having the capacity to answer questionnaire within 20 min and 5) agreeing to participate. A convenient sampling technique was used to enroll a total of 1016 patients in this study. Patients were invited into a designated room for face-to-face interviewing. Before the interview, participants were introduced to the study and asked to provide written informed consent. The response rate was 80–90 % across sites. Interviewers were master’s students of public health at Hanoi Medical University. The students were working in HIV study and had no affiliation with the clinics in which they invited patients to participate.

### Measures and instruments

A structured questionnaire was developed to use in this study. Data on socioeconomic characteristics, drug use behaviors, HIV status was interviewed. Drug use information included age at initial drug use, time since first drug injection, times of previous drug rehabilitation, and duration of MMT treatment. Health status of respondents was measured in five dimensions (mobility, self-care, usual activities, pain/discomfort, and anxiety/depression) using the five-level EQ-5D (EQ-5D-5 L) instrument that has been validated and widely used in Vietnamese populations [6, 27, 28]. Responses were then recorded to the EQ-5D dimensions as either having any health problems or no problems.

It has been well-documented that the stigma against HIV and drug use in Vietnam has been fueled by both the fear of HIV infection and social values and by judgements on addiction and other risk behaviours [3, 18, 29–32]. In some settings that contain large drug-using populations with generalized HIV epidemics, the stigma and discrimination against HIV/AIDS and addiction are intertwined [3, 33, 34]. We referred to the Substance Abuse Self-Stigma Scale by Luoma and measures of HIV-related stigma [35, 36]. In addition, we adapted the conceptual framework by Parker and Aggleton to construct the measure of drug addiction-related stigma among MMT patients [37]. We then piloted the measures in drug users and people living with HIV/AIDS. The final measure of stigma included five dimensions: (1)Blame/Judgement, (2) Shame, (3) Discrimination in various settings (work place, health care services, family, and community), (4) Disclosure of addiction or health status (including HIV-positive if seropositive), and (5) Other’s fear of HIV transmission among those patients who self-reported being HIV-positive [36]. Respondents were asked if they had experienced any of the above types of stigma within the last month. The measure, for example, included the following questions with the response options: Yes/No/Not answer.

1. In general, have you recently been blamed or criticized because of your health status and drug use behaviors?
2. Do you currently feel shame because of your health status and drug use behaviors?
3. Have you felt discriminated against or treated badly by others? In which circumstances (work place/all health facilities/family/community/others)?.
4. Have you ever disclosed your health status and drug use behaviors with others? With whom did you share?
5. (For HIV positives) Has anyone expressed fear of contracting HIV from casual contacts with you?

### Statistical analysis

*T*-test and χ2 tests were used to compare differences of characteristics among different services models. Multivariate logistic regression was employed to determine the associated factors with self-stigma, discrimination, and disclosure. In this study, a stepwise backward selection strategy was applied along with multivariate regression to have reduced models. This strategy used threshold with the log-likelihood ratio test to have predictors with *p*-values of < 0.1 included.

### Ethics, consent and permissions

The protocol of this study was reviewed and approved by the Vietnam Authority of HIV/AIDS Control's Scientific Research Committee. Written informed consent was obtained from all participants. Patients could withdraw at any time without the influence on their current treatment.

## Results

In total, 1016 patients participated in this study; 746 were receiving MMT at one of four DHCs, and 270 others were receiving MMT at Nam Dinh PAC. Among those, 98.7 % were male and the mean age was 36.8 years (SD = 7.7). The majority lived with a spouse or partner (67.7 %) and had high school education or below (93.4 %). The percentage of patients who were currently working was 74.6 %, and of these, 53.4 % were self-employed, 9.8 % were workers or farmers, and 11.4 % had other jobs (white collars, students, and other) (Table 2).

**Table 2:** Socioeconomic characteristics of MMT patients by level of health services administration

	Provincial		District		All		*p*-value
	Mean	SD	Mean	SD	Mean	SD	
Age	36.8	7.7	36.8	7.7	36.8	7.6	0.52
	N	%	N	%	N	%	
Sex (Male)	266	98.5	737	98.8	1003	98.7	0.73
Educational attainment							
Illiterate	4	1.5	13	1.7	17	1.7	0.03
Elementary	21	7.8	98	13.1	119	11.7	
Secondary	103	38.2	323	43.3	426	41.9	
High	121	44.8	266	35.7	387	38.1	
Vocational	12	4.4	20	2.7	32	3.2	
University	9	3.3	26	3.5	35	3.4	
Marital status							
Single	101	37.4	150	20.1	251	24.7	<0.01
Live with spouse	148	54.8	537	72.0	685	67.4	
Live with partner	0	0.0	3	0.4	3	0.3	
Divorced	19	7.0	53	7.1	72	7.1	
Widow	2	0.7	3	0.4	5	0.5	
Religion							
Cult of ancestors	247	91.5	649	87.0	896	88.2	0.17
Buddhism	13	4.8	46	6.2	59	5.8	
Catholic	10	3.7	46	6.2	56	5.5	
Protestant	0	0.0	5	0.7	5	0.5	
Employment							
Unemployed	76	28.2	183	24.5	259	25.5	<0.01
Self-employed	159	58.9	383	51.3	542	53.4	
White collars	5	1.9	17	2.3	22	2.2	
Workers, Farmers	10	3.7	90	12.1	100	9.8	
Students	0	0.0	2	0.3	2	0.2	
Other jobs	20	7.4	71	9.5	91	9.0	

Table 3 presents health status, history of drug addiction, and MMT utilization of participants. The average age of drug use initiation was 24.5 years (SD = 6.7), corresponding to an average duration of drugs use of 13.3 years (SD = 5.9) and drug injection duration of 10.2 years (SD = 4.9). Enrolled patients experienced approximately 5 episodes (mean = 4.8) of drug rehabilitation prior to MMT. The duration of MMT utilization was 16.5 months on average (SD = 11.0); patients from PAC experienced an average of 11.4 months (SD = 7.2) while patients from DHC had undergone a longer duration on MMT (mean = 18.4 months, SD = 11.5). Regarding health status of patients, we found homogeneity in outcomes between provincial and district health facilities, except a higher prevalence of pain or discomfort among patients at DHCs. The prevalence of patients who reported any health problem was highest in the anxiety/depression dimension (20.7 %), followed by pain/discomfort (19.2 %).4.8 % of patients reported concurrent drug use during MMT and the percentage of self-reported positive HIV status was 8.1 %.

**Table 3:** Health status and history of drug addiction and MMT

	Provincial		District		All		*p*-value
	Mean	SD	Mean	SD	Mean	SD	
Age at first drug use	24.3	7.0	24.6	6.7	24.5	6.7	0.10
Time since 1st drug use (yr.)	13.5	5.1	13.2	6.1	13.3	5.9	0.07
Time since 1st drug inject(yr.)	10.9	4.4	9.9	5.1	10.2	4.9	0.12
# previous drug rehabilitation episodes	5.7	7.2	4.5	5.9	4.8	6.3	<0.01
Duration on MMT (month)	11.4	7.2	18.4	11.5	16.5	11.0	<0.01
	N	%	N	%	N	%	
Self-reported health problems							
Mobility	19	7.04	55	7.37	74	7.28	0.86
Self-care	10	3.7	30	4.02	40	3.94	0.82
Usual Activities	18	6.67	42	5.63	60	5.91	0.54
Pain or Discomfort	37	13.7	143	19.17	180	17.72	0.04
Anxiety or Depression	56	20.74	154	20.64	210	20.67	0.97
Concurrent drug use	15	5.56	34	4.56	49	4.82	0.51
History of drug injection	222	82.22	524	70.24	746	73.43	<0.01
Self-reported HIV status		0		0		0	
Negative	239	88.52	637	85.39	876	86.22	0.15
Positive	22	8.15	60	8.04	82	8.07	
N/A	9	3.33	49	6.57	58	5.71	

In Table 4, the proportion of stigma and discrimination among MMT patients is shown. More than one-sixth of the sample population reported experiencing different types of stigma or discrimination related to their addiction or health status, including Blame/Judgement (17.2 %), Shame (19.9 %), or Fear of HIV transmission by others (17.1 %); these percentages were higher in PAC than in DHCs. There were very few patients who reported discrimination at the workplace (2.5 %) or at health care services (1.7 %); however, 15.6 % patients at PAC and 10.6 % patients at DHCs reported discrimination from their communities. The proportion of respondents who disclosed their addiction or health status to others was low (with spouse (58.3 %), parents (50.4 %), health workers (37.3 %), and peer educators (14.3 %)). Patients who were taking MMT at DHCs reported less discrimination and more disclosure of their status to others.

**Table 4:** Proportion of stigma and discrimination among MMT patients

	Provincial		District		All		*p*-value
	N	%	N	%	N	%	
1. Blame, judge	46	19.7	119	16.4	165	17.2	0.25
2. Shame	66	28.5	124	17.2	190	19.9	<0.01
3. Discrimination							
Work place	12	4.4	13	1.7	25	2.5	0.01
Health care services	9	3.3	8	1.1	17	1.7	0.01
Family	8	3.0	16	2.1	24	2.4	0.45
Community	42	15.6	79	10.6	121	11.9	0.03
4. Disclosure							
Spouse	109	40.4	483	64.8	592	58.3	<0.01
Parents	133	49.3	379	50.8	512	50.4	0.66
Relatives	53	19.6	229	30.7	282	27.8	<0.01
Friends	59	21.9	237	31.8	296	29.1	<0.01
Health workers	72	26.7	307	41.2	379	37.3	<0.01
Peer educators	38	14.1	107	14.3	145	14.3	0.91
5. Others’ fears of HIV	5	22.7	9	15.0	14	17.1	0.41

Table 5 presents the factors that are associated with stigma and discrimination against drug users. Drug users taking MMT for longer periods were less likely to report being blamed or judged (OR = 0.98, 95 % CI = 0.97–0.99), but they were also less likely to disclose their health condition to others (OR = 0.96, 95 % CI = 0.95–0.98). A higher likelihood of disclosing health conditions was associated with people living with a spouse (OR = 1.56, 95 % CI = 1.14–2.15) versus patients living alone. Other factors that contributed to perceived stigma and discrimination among MMT patients included higher education, presence of pain/discomfort and anxiety/depression, self-reported HIV positive, and number of previous drug rehabilitation episodes. Moreover, these factors also increased the likelihood of disclosing addiction and any health problem among MMT patients.

**Table 5:** Factors associated with stigma and discrimination against drug users

	Blame, judge			Shame			Discrimination			Disclosure of addiction or health status		
	OR	95 CI		OR	95 CI		OR	95 CI		OR	95 CI	
Duration on MMT (months)	0.98^a^	0.97	0.99	1.00	0.98	1.02	0.99	0.96	1.01	0.96^a^	0.95	0.98
MMT model (MMT+ PAC - ref)												
Rural MMT-ART-VCT-DGH	1.67	0.99	2.82									
Urban MMT-ART-VCT-DGH				0.36^a^	0.22	0.58	0.51^a^	0.31	0.84	3.21^a^	2.13	4.83
MMT + Regional poly clinic				0.39^a^	0.24	0.63						
Education (High school or Lower - ref)												
College/University	1.82	0.93	3.57				1.96^a^	1.02	3.74			
Marital status												
Live with spouse vs. Single										1.56^a^	1.14	2.15
Religion												
Buddhism vs. Cult of ancestors	0.43	0.15	1.22									
Employment (Unemployed - ref)												
Self-employed	0.72	0.49	1.04									
White collars	0.30	0.06	1.44									
Workers, Farmers	0.42^a^	0.19	0.93	0.50^a^	0.25	0.99	0.52	0.24	1.13	0.49^a^	0.30	0.80
Other jobs				1.65	0.93	2.95						
Self-reported health problems												
Mobility				1.82	1.00	3.31				0.39^a^	0.20	0.74
Self-care							2.12	0.94	4.81			
Usual Activities										2.22	0.90	5.47
Pain or Discomfort										2.01^a^	1.16	3.47
Anxiety or Depression	1.79^a^	1.19	2.70	2.39^a^	1.62	3.53	1.49	0.95	2.32	1.66^a^	1.02	2.69
Self-reported HIV status (Negative - ref)												
Positive				1.74^a^	1.01	3.04						
N/A							2.15^a^	1.04	4.47			
# previous drug rehabilitation (None - ref)												
1–5 times	2.27	0.88	5.88				0.73	0.49	1.08	1.86^a^	1.21	2.86
6–10 times	2.89^a^	1.07	7.80							1.73^a^	1.03	2.89
> 10 times	2.75	0.85	8.83									
Ever inject drug vs. None							1.53	0.94	2.48			
Concurrent drug use vs. None							2.44^a^	1.11	5.37			

^a^significant at 5 % level

## Discussion

To date, this is the first study comparing drug addiction-related stigma among MMT patients across different service delivery models and levels of administration. The findings showed that though MMT has been known to improve health behaviors among drug users, a high proportion of users suffered from stigma and discrimination due to their previous drug addiction or health status. Although felt stigma decreased among patients who were enrolled in MMT for longer periods and discrimination was rarely seen in health facilities, workplace, and family life, felt stigma remained high in communities where patients live, especially in urban areas and in areas of higher level of health care service administration.

### Stigma in relation to MMT and different service delivery models

Our findings highlight the encouraging progress in quality improvement and stigma reduction in HIV-related health care services in Vietnam. Previously, stigma was commonly reported in health care services, which reduced the access and use of health care and support services among people with HIV/AIDS [18], and was burdensome to health workers in HIV facilities [30]. In this study, just about 2 % of respondents reported experiencing any discrimination at health care services. Meanwhile, the high level of discrimination from communities against drug users is consistent with a previous qualitative analysis in Thai Nguyen Province by Rudolph et al. [31] In addition to previous studies, we found that not only socioeconomic status and history of drug rehabilitation significantly predicted stigma and discrimination among drug users taking MMT, but also other major factors included health status and the availability of comprehensive HIV/AIDS and general health care services.

In Vietnam, a DHC is the closest health care facility providing methadone medication for drug dependence treatment. In this study, patients at DHCs or regional polyclinics were less likely to be stigmatized compared to those at PAC. One study by Mukora et al. showed that patients were concerned about stigma and loss of privacy if they received treatment in decentralized clinics [38]. However, a study by Odeny et al. found that decentralized HIV-related services that were integrated into primary health care did not worsen stigma [39]. The differences in patients’ preferences and perceived stigma highlighted the importance of understanding contextual factors and stages of the epidemics in each setting. In this study, fewer patients felt ashamed or experienced discrimination at MMT clinics with other HIV-related and general health care services; nonetheless, these results may not apply to those living in rural areas.

In addition, we found that drug users taking MMT for longer periods of time felt less blamed or judged (OR = 0.98, 95 % CI = 0.97–0.99). This may be due to the effects of MMT on the improvement of health status and the reduction of risk behaviors and illegal activities, which may promote positive attitudes of family and others toward MMT patients. Previous studies have shown that MMT patients improved substantially in physical health; however, the changes in mental and social status were moderate and small, respectively [7, 40]. Participants reporting anxiety and depression were also much more likely to report feeling blamed/judged and shame. In addition, drug users who currently used drugs during MMT were more likely to suffer from discrimination than those who did not. Collectively, these results indicated the importance of maintaining MMT to reduce drug use behaviors, as well as the importance of providing comprehensive mental health care for patients.

The study also suggests that the use of MMT is preferred more than traditional drug rehabilitation in Vietnam. Less effective drug rehabilitation was significantly associated with higher stigma. Drug detoxification is available in Vietnam [6]^,^ but with high episodes of failure rehabilitation, patients may believe that they cannot be successfully treated, and therefore, blame themselves for relapsing. In this study, the proportion of felt stigma was lower than in other settings. This coincides with findings from previous studies that MMT helps improve social and economic status of patients and their families [5, 7, 40–42].

Notably, the proportion of MMT patients’ felt stigma in communities was significantly higher than in families, health facilities, or workplaces. This result suggested that although participating in MMT program could reduce stigma in those latter place, stigma remained a great barrier for MMT patients trying to reenter into their community. A study conducted by Tomori et al. in Vietnam showed that even when a person successfully detoxifies from illicit drugs, they had to confront the drug-related stigmatization in their community [2]. Therefore, mass media campaigns are necessary to reduce stigma against people with HIV/AIDS and history of drug addiction in settings with large drug injection related HIV epidemics.

In this study, we also found that patients who were HIV positive or unknown status were more likely to report self-stigmatization. Literature suggests that drug users report shameful attitudes and think that HIV might be a punishment for their past [2]. On the other hand, it is noteworthy to find that people who were employed were less likely to feel self-stigmatization. Vocational training and job referrals should be considered in local planning to maximize the benefits of MMT programs.

### Disclosing MMT patients’ status

Disclosure of status among HIV-positive people and PWID has been considered a potential method to reduce the spread of HIV/AIDS. Although the drawbacks of self-disclosure may include stigma, loss of privacy, and blame, it may help those high-risk populations to reduce HIV risk behaviors and increase access to HIV-related care, as well as being related to better adherence for those receiving treatment [18]. In present study, patients receiving MMT at DHC reported a higher percentage of disclosing their condition to spouses, relatives, health workers, and friends than patients at PAC. This association can be partially explained by the fact that in order to hide their conditions and avoid discrimination, drug users tended to receive treatment at a health center far from their hometowns [43].

People taking MMT in long duration were less likely to disclose their health status to other people. There may several reasons be for this finding. First, as we observed, with the improvement in health status due to long duration of treatment, MMT patients might feel confident about their health and therefore may think that they do not need to share their status with other people. Second, information about MMT patients’ treatment or their past may be used against them at work or in the community [44].

Most patients shared their status with their spouse (58.3 %) and parents (50.4 %), who were the closest relatives of patients. People living with a spouse also were likely to disclose. Families are a critical source of financial, physical, and emotional supports, which facilitate re-entry into the community for drug users [45]. Therefore, support from family has a central role in encouraging patients’ disclosure.

### Implications

There were several implications that can be drawn from this study. First, MMT clinics should be integrated with other health services and decentralized as satellite model to provide patients with accessible and friendly health care, which in turn may reduce stigma of patients. Strategies to optimize the effectiveness of this model should be considered for the scale-up plan of MMT programs. Second, mass media campaigns in television or on the internet should be conducted to reduce stigmatization in the community for MMT patients. Third, the role of parents/spouses should be heightened to help combat difficulties encountered when patients participate in MMT treatment. Lastly, physical and mental health conditions of patients should be acknowledged and addressed.

### Limitations

Several limitations in this study should be under consideration. First, this cross-sectional design could not establish causal relation between stigma outcomes and MMT delivery models. In addition, a qualitative study should be conducted to understand how different models can impact on the feeling of stigma in accordance to patients’ perceptions and experiences. Second, Self-report information may be subject to recall bias. Finally, the convenient sampling technique limited the generalization of this study.

## Conclusions

In conclusion, the study highlighted significant higher levels of stigma among MMT patients at PAC as compared to people at DHC. The findings suggested the need to integrate MMT with the satellite model (DHC, regional poly-clinics, etc.) to reduce stigma. Moreover, the study emphasized the importance of maintaining MMT adherence and effectiveness and the importance of intervening on stigma amongst drug users, family, health care workers, and community.

### Availability of data and materials

Data are available from the Authority of HIV/AIDS Control (VAAC). However, since the Government of Vietnam issued the Law on HIV/AIDS, information of HIV-affected people is confidential and cannot be shared. Requests for data on this study may be submitted to VAAC and go through a review process by the Scientific and Ethical Research Committee. The contact for requesting data use is Dr. Phan Thi Thu Huong, email huongphanmoh@gmail.com, Deputy Director in Research of the Vietnam Authority of HIV/AIDS Control, Ministry of Health, Vietnam.
